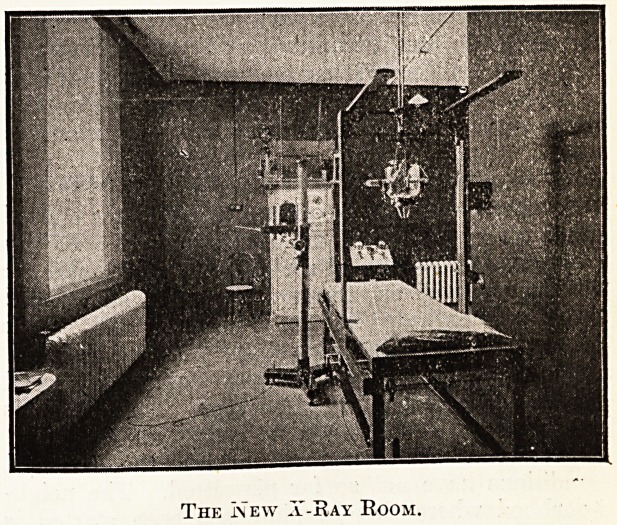# Hospital and Institutional News

**Published:** 1913-08-16

**Authors:** 


### HOSPITAL AND INSTITUTIONAL NEWS.

### MR. BURNS ON INSTITUTIONAL LIFE.

The final general address at the International Medical Congress, which Mr. John Burns delivered at the Albert Hall on Tuesday, was exceedingly happy in that its subject and treatment gave a practical ending to the previous, sometimes theoretical and sometimes practical, debates. Nothing better illustrates the important part which Mr. Burns' personality and Department play in the health of the nation than the fact that he is the first Minister of State, throughout all the seventeen Congresses, to take a vital part in its regular sessions. As the responsible head of the Department to which the public health of the nation is entrusted he was able to trace the increasing importance of institutional life and institutional methods in the treatment and prevention of disease. Not long ago we published a remarkable interview with the medical superintendent of a Poor-Law infirmary, in which were described the relation and value of the Government inspectors to these institutions and to their managers. Mr. Burns knows well enough the value of the Departmental inspectors to his administration, and nothing was more striking than the way in which the infirmary superintendent alluded to, recorded his opinion how the value of the inspectors to the infirmaries might be increased, and how more numerous inspections would be welcomed. The institution, which is now becoming the trusted ally of preventive medicine in its widest and most statesmanlike aspects, has now to be correlated to its fellows, as are the ganglia of a nerve, separate yet related. Mr. Burns was right to point out what the nation owes to institutional methods.

### PIONEERS OF PUBLIC HEALTH.

"The benefit," Mr. Burns continued, "of many of the large capital expenditures on hospitals, housing, water supplies, sewerage, would be reaped even more in the future than in the record of the past." He divided the progress in health at different periods into two processes: first, the crude generalisations in favour of sanitary reform; and, second, when the attack on each disease was based on the results of scientific investigation. We are glad that he recalled these voluntary workers, the pioneers of public health, hospital, and sanitary reform, John Howard, Elizabeth Fry (whose defects were almost as inspiriting as her qualities), Sadler, Shaftesbury, Charles Dickens, Charles Kingsley (who attacked abuses with the mighty weapons of reiteration and laughter), and in official life, the great Edwin Chadwick of the Poor-Law Board. For him Mr. Burns had a special tribute: "It was not the least of Chadwick's great services to the State in Great Britain that he discovered William Farr, the humble apothecary, and placed him in charge of our national vital statistics. The later reports by Drs. Ogle, Tatham, and Stevenson had worthily continued the series initiated by Farr." And he did not forget to record the services of medical men. Pioneer attempts made by medical men in a number of districts to secure the voluntary notification of different forms of illness had led, he remarked, to agitation in favour of the compulsory notification of the chief acute infectious diseases.

### THE FUNCTIONS OF SANATORIA.

Taking tuberculosis as the most important instance of the extension of the principle of notification, Mr. Burns referred to the work of Semmelweiss, Lister, Koch, and Pasteur, and remarked upon the steady increase in the use of institutions for the tuberculous sick. The figures which he then gave are worth recording in full. "In England and Wales in 1911 34 per cent. of the male and 22 per cent. of the female deaths from phthisis, and in London 59 per cent. of the male and 48 per cent. of the female deaths, occurred in public institutions. The patients had usually spent several months in them prior to death. By this means, therefore, not only had the families of the patients been relieved of a serious burden, but also had been released from the risk of infection at a time when this was especially difficult to control." This last point was worth making, because we so often hear it argued as if the test of the success of any treatment, particularly sanatorium treatment, were merely the percentage of cures. Hence their hasty rejection sometimes of all but picked cases. The sanatoria which have accepted advanced cases—still to-day they often hardly hear of them before that stage is reached—have also done immense service in controlling the centre of infection which an advanced case certainly is.

### BETTING IN POOR-LAW INFIRMARIES.

Improvements in Bermondsey Infirmary are being carried out with rapidity, partly due to the advent on the Board of Dr. A. Salter. In the dietary table he has induced his colleagues to substitute butter for margarine, and to engage wardmaids in lieu of casual scrubbers. The latter have led to many abuses, which have proved injurious to the patients and the general discipline of the institution. The regulations forbidding them to introduce articles of food, wines, spirits, or intoxicating liquors were inoperative, and, according to a report prepared by the Clerk, betting transactions took place in the institution, the women being the means of communication with bookmakers outside. The employment of a large number of irresponsible women as scrubbers entailed upon the medical staff and the matron and her assistant a large amount of unnecessary work and anxiety, and these difficulties were increased by dislocation of the work through the absence, often without notice, of the scrubbers. The result was that the work was not satisfactorily performed, and even after the maximum of effort to get the work done, it often ended in failure. Acting on the advice of the Clerk, supported by Dr. Salter, the Board resolved to discontinue the employment of what was described as "casual labour," and to engage in its place ward-helpers, who will devote their whole time to the duties six days a week.

### HOSPITAL MANAGEMENT IN CATHEDRAL CITIES.

What is it that causes the standard of administration of voluntary hospitals situated in cathedral cities to be frequently so far below the standard of a modern hospital in these islands? Is it due to the fact that the situation of several cathedrals is such as to diminish energy for climatic reasons amongst the inhabitants? A relaxing climate and a sleepy population are not infrequently closely associated. Whatever it be, no trained observer with expert knowledge who made it his business to inspect and study the working of the voluntary hospitals in cathedral cities throughout England could fail to be struck with the absence of knowledge and keen administrative ability which some of them present. Indeed, the administration in some is so faulty as to be lamentable, as our columns have demonstrated in recent years. The state of affairs revealed first at Chester, then at York, astonished the country; and although Chester has bestirred itself and is making strong efforts to provide an up-to-date modern hospital, York appears to be so immersed in indifference that, despite the report and findings of the Commission, the administration there seems to remain asleep, and the citizens of York well content to have it so. Yet York should be provided without delay with a modern, up-to-date hospital, for the average prosperity of its citizens is high, and the great cocoa magnates who rule its destinies could well afford to give York a new hospital placed upon an adequate site, if they had the same public spirit as that exhibited in so many of our cities by other wealthy manufacturers and capitalists. In this connection it is just to say that the managers of the Salisbury Infirmary, the Gloucestershire Royal Infirmary, and the Norfolk and Norwich Hospital have brought the voluntary hospitals in these cathedral centres to a high state of efficiency. What they have done other cathedral cities may properly be called upon to do also. If the citizens of Worcester would pay a visit to Gloucester and learn how a voluntary hospital can be wisely administered and successfully financed, we have little doubt that they would speedily insist that the Worcester Infirmary should without further delay be converted into a prosperous, ably administered, and successful modern hospital. How true it is that the inhabitants of a city have the hospital which from their intelligence and energy or the absence of these qualities they deserve!

### RESTARTING A COTTAGE HOSPITAL.

Our advice in a recent issue to the governors of Morpeth Cottage Hospital appears to have borne fruit, for the amendment of Dr. Philip, urging that the institution should be carried on by a new committee, which was passed by a narrow majority, has been followed up by an extraordinary meeting at which a new committee was appointed. In view of our remarks that the difficulties alleged by Canon Davis to exist owing to a want of co-operation on the part of the doctors in the town should be removed by a joint conference, it is interesting to add that Dr. Philip in commending his scheme for a new committee to the meeting stated in so many words that it had met with the unanimous approval of the other medical practitioners in the town. The scheme, which was carried unanimously, had the effect of appointing a committee of management consisting of six ladies to represent the town and two to represent the country. It also gave this committee power to appoint a secretary. A sign of hopefulness that the new committee may prove more successful than its predecessor may be seen in the fact that the hospital's president, Mrs. Blencowe Cookson, though now living out of the immediate locality, is continuing her association with it. Till a new secretary be appointed, Mr. T. Hopper is continuing to act.

### MEDICAL APPOINTMENTS TO THE LEGION OF HONOUR.

On account of the Pasteur celebrations, many medical names are included in the appointments to and promotions in the Legion of Honour announced this week. Dr. Roux, the director of the Pasteur Institute, is appointed Grand Officer, and among the new Commanders noted by the foreign correspondent of the *Times* are Dr. Metchnikoff, the assistant director of the Institute, and Drs. Yersin, Faisans, Pouchet, Reclus, and Pitres. The new Officers of the Legion include Professor Bouvier, M. Vallery-Radot, the biographer of Pasteur, M. Bottel, and Dr. Pierre-Marie. There are said to be fifty doctors in the list.

### DOCTOR REFUSES TO INSURE HIS SERVANTS.

Mr. E. H. Squire, M.R.C.S., a practitioner of Wivenhoe, in Essex, was fined last Saturday an aggregate of some £7 for refusing to pay contributions under the Insurance Act for two servants. Mr. Squire declared that he considered that the Insurance Act was an imposition upon the King's subjects, and that as a country practitioner who had to mix with the working classes he knew the Chancellor of the Exchequer's statement that the people liked the Insurance Act was untrue. He added that from the first he had refused to join the panel, and that only himself and two practitioners at Colchester had kept their word. One of the reasons why the scarcity of medical members of Parliament is to be regretted is that the medical profession has such peculiar opportunities for knowing the minds of voters on questions of this kind.

### CHRISTIAN SCIENTIST CHARGED WITH MANSLAUGHTER.

As the result of an inquest on the child of a Christian Scientist, a man named Jewell has been committed for trial on a charge of manslaughter. The case was one of the usual kind. As a result of a post-mortem examination, a medical man said that diphtheritic germs were found in the child's throat. When he was called in the child was dead, and its parents seemed to think that it had had mumps. He thought, but he could not be certain, that death was due to diphtheria. He also thought that medical aid should have been called in. Dr. John Macpherson Johnston, assistant medical officer of health for the Borough of Hornsey, stated his opinion that 30 per cent. of cases having similar post-mortem appearances would die. The coroner said that there now could be no doubt that the child died of diphtheria. He pointed out that legally it did not matter whether or not in refusing to call in a doctor the parents were influenced by conscientious or religious motives. Twelve out of the thirteen jurors, in answer to the coroner's questions, declared that in their opinion a reasonable person would have called in a doctor, and that death was accelerated by the want of medical aid. Bail was offered and forthcoming.

### SUMMER WILD FLOWERS FOR HOSPITALS.

There is a great profusion of wild flowers in the late summer and early autumn, and ample leisure to gather them when away on a holiday. Such wild flowers are very welcome in the hospitals. The cost of transmission is inconsiderable, if they be sent by post or rail; and, if due care be exercised, they are pretty sure to arrive in a fresh or comparatively fresh condition. Many varieties of summer and early autumn wild flowers are of a hardy kind, and would bear the journey well. We may instance the ox-eye daisy, the handsome yellow ragwort and fleabane, purple loosestrife, the hairy willow-herb, with its pinky blossoms and white central disc, and several varieties of heather. The purple heather is very plentiful on our moors and heaths. Moreover, there are sea-lavender and sea-holly which grow profusely on many of our coasts, and last so long either in water or otherwise when gathered. Teasels and feathery rushes and long grasses make pretty decorations. We are sure that many of our readers will not be slow to take the hint which we have pleasure in offering.

### THE MATERNITY BENEFIT MANŒUVRES.

Fate is jealous of her ironies. The amendment moved by Mr. Roberts, by which *either* the wife's or the husband's receipt for the maternity benefit that is payable in respect of the husband's insurance should be sufficient discharge, was actually passed. In spite of the further pious phrase that the husband should pay it over to the wife, the wife's security, as we pointed out last week, is thus gone, and, in the words of Lord Robert Cecil's capital speech, the clause that "maternity benefit shall in every case be the mother's benefit" was rendered valueless. This, too, in Congress week, and, last sting of all, on a question which was left open by the Government, and for which the Party Whips did not act as tellers. But in Lord Robert Cecil the mothers had a champion who insisted on having the last word, and his proposal that the husband's receipt for the payment of the benefit should only be valid when he has been authorised by the wife to receive the money, which was a noble attempt to undo the mischief just accomplished, was carried. The votes were 186 to 165, and so the mothers' majority of 21 may really get them their 30s. after all.

### THE INTERIOR RECONSTRUCTION AT CHICHESTER.

The fine illustration which we published last week of the Royal West Sussex Hospital, showing the new out-patient unit on the right hand of the photograph, gave a capital idea of the extension, but, of course, could not illustrate the interior reconstructions that have played an equally important

The Interior of the William James Ward.

part in the scheme. We publish this week, therefore, an illustration of the William James Ward, showing its typical shape, design, and fittings, and also, to give another instance of the reconstruction, a photograph of the new X-Ray Room. Both photographs are by W. P. Marsh and Sons, Chichester.

The New X-Ray Room.

The relative size of the new X-Ray Room is particularly to be noted, since such rooms are frequently required before there is the money to build them, and as often as not are little more than cupboards, and even when specially built are sometimes unduly cramped.

### WESTMORLAND SANATORIUM ENTERPRISE.

The county of Westmorland has included, we understand, in its ultimate scheme for the provision of sanatorium benefit under the National Insurance Act the establishment of an Open-air Recovery School, to be erected in the grounds of the Westmorland Consumption Sanatorium, for the institutional treatment of school children suffering from tuberculosis. This Sanatorium, under the energetic management of Drs. Rushton Parker and Paget Tomlinson, has already fairly recently provided a home for advanced cases of consumption, which we believe is successfully conducted in connection with the ordinary work of the institution. It has also been extended to meet the present demand for beds for consumptives. This further evidence of enterprise is gratifying, and, coming as it does from a thinly populated locality, should put to shame the comparative inaction of some Sanatorium authorities in large cities. Probably the real reason of this last phenomenon is the reflection that to enlist fresh support means conceding fresh representation in the management. But, although a little nervousness and jealousy of eleventh-hour arrivals in the anti-tuberculosis field may be what is to be expected of human nature, yet it is surely not to be allowed for a moment to hinder possible development of the sphere of an institution's work. Moreover, it is not fair to institutional employees. Those who have worked in the past under unavoidable limitations and disabilities should, now that the chance of better times has come, be allowed to profit by it. The interests of the patients themselves—the paramount consideration, of course—will certainly be better served by experienced people than by those who two or three years ago had never thought of the subject of tuberculosis at all. Financially and in every way, extensions of existing centres of activity are much to be preferred to the hurried initiation of new institutions. Yet, if committees of management deliberately keep to an anti-progressive attitude, they will have only themselves to thank if they suffer supersession.

### THE WOMEN'S HOSPITAL FOR CHILDREN.

A year or two ago a committee of ladies started a clinic for children in the Harrow Road. To this institution the name of the Women's Hospital for Children was given, to show that it is staffed and organised entirely by the feminine sex. The other day an open-air demonstration was arranged, and was supported by local trade unions and social bodies of the labouring-class type. The object is to raise sufficient funds to start an in-patient department, a development which the financial conditions have not so far permitted. The neighbourhood where this hospital has been started is very poor indeed, and the nearest children's hospital is Paddington Green—by no means inaccessible, but some little distance away. It is therefore not surprising to find that the clinic is abundantly supplied with material, and could do more good if an in-patient department existed. Indeed, this deficiency seriously detracts from the usefulness of the out-patient department. Take, for instance, the case of such a common condition as tonsils and adenoids. The operation for this is, we believe, often done at the Harrow Road clinic; the patients, of course, being taken home by their parents afterwards. Sooner or later the inevitable case of severe hæmorrhage will occur; and then the patient will suffer from the lack of a bed to be put into for a day or two. And there are many other "out-patient operations" which are legitimate enough at an institution with facilities for looking after the thousandth case when some emergency arises, but are morally indefensible where no such accommodation exists.

### THE KING AND HOSPITAL PROGRESS.

The King, who is patron of St. George's Hospital and one of the patrons of Westminster Hospital, has commanded that a letter should be written stating that their Majesties were most gratified to learn of the proposed amalgamation of the two institutions, and placing on record their opinion that the amalgamation is likely to be "for the greater benefit of the sick and suffering poor." Every important development in the voluntary hospital system ever since the inauguration of the Hospital Fund for London has been brought about and encouraged by Royal initiative and support; and it is only fitting therefore that this latest movement should receive public record of the King's approbation.

### THE MENTAL DEFICIENCY BILL.

In the House of Lords, on Tuesday, after various amendments, which had appeared on the Order Paper under the Lord Chancellor's name, had been agreed to, the Mental Deficiency Bill having been read a third time, as amended, was passed.

### LARGE SHELTERS FOR HOLIDAY CHILDREN.

So many thousands of holiday children are taken on day excursions to the seaside and other places where they are exposed to the vicissitudes of the weather, that the suggestion might be offered that where shelters of a considerable size do not exist, it would be well to provide them. Fortunately, in some directions this has been done; in others, if the day should turn out showery or be persistently wet when the children have arrived at their destination, they often have a very sorry time. They are usually lightly clad in summer attire, which soon becomes saturated with wet, for the majority of the little ones come ill-provided with or without wraps. The consequence is that they spend anything but a happy day in their bedraggled condition, and return home in a comfortless state in their wet or partly wet clothes, and a very serious risk to health is incurred. Large shelters could be provided at no considerable cost, preferably on, or near, the sea-frontage, where the children could get glimpses of the waves, and engage in their games well sheltered from the wind and rain. Shelters of this sort could also be erected with great benefit to the children on some of the London and other commons and open spaces.

### THE DRAFT POOR-LAW INSTITUTIONS ORDER.

The first Report of the Departmental Committee appointed by the President of the Local Government Board with respect to Poor-Law Orders is published this week. It contains, of course, only tentative proposals, which, as such, are not at present suitable for publication *in extenso*, but in so far as they indicate the trend of approaching changes, and the lines on which the authority of the medical and nursing officials is likely to be consolidated or developed, our readers will appreciate a summary and criticism of the Report wherever its provisions are likely to affect the future and status of the Poor-Law infirmary and its officers. We propose, therefore, to discuss the Report at the earliest moment from the point of view of the best interests of the Poor-Law infirmary.

### DEWSBURY INFIRMARY AND THE INTERNATIONAL MEDICAL CONGRESS.

At the monthly meeting of the board of management of the Dewsbury and District General Infirmary held on Tuesday evening, the 12th inst., Mr. Blackburn (Heckmondwike), president, in the chair, the following motion, proposed by Mr. W. W. Yates, seconded by Mr. R. Armstrong, and supported by Mr. H. Thorp, was unanimously adopted, viz.: "That in order to mark the high appreciation the board entertains of the continued valuable services of the honorary medical staff of this infirmary, and at the same time to render their services in the future still more valuable, it is hereby resolved to purchase as soon as issued, the volumes containing the Official Report of the proceedings of the International Medical Congress now being held in London; and that the house committee be empowered to obtain a suitable bookcase for the reception of the said volumes, if one is found to be required."

### A NEW MATRON AT CAMBRIDGE.

Miss Constance C. Crookenden, at present matron of the Cray Valley Hospital, St. Mary Cray, Kent, has been elected matron at Addenbrooke's Hospital, Cambridge, in succession to Miss M. G. Montgomery, who was recently appointed lady superintendent of the Middlesex Hospital. After training and holding appointments at St. Thomas's Hospital, London, Miss Crookenden was appointed to her present post in 1908. It is expected that the new matron will take up her duties at Addenbrooke's early in October.

### "INSTRUCTIVE MISTAKES."

A short time ago we welcomed the announcement of the impending issue of a new quarterly magazine, and now the first number lies before us. Excellently printed and produced in every way, the *British Journal of Surgery* will stand comparison with any of the Continental and American magazines devoted to the subject. Besides the original articles, which are of a high standard of merit, there are several further features which arrest attention. The two admirable "critical reviews" on Surgical Shock and Recent Methods of Anæsthesia respectively are not a new idea; for several of the best class medical journals publish articles of similar scope. "Instructive Mistakes" is, however, more by way of a novelty: it is an anonymous section, containing brief reports of cases of mistaken diagnosis and of the reasons for the errors. Descriptions of visits to well-known surgical clinics at home and abroad are another valuable feature; these are written in a candid vein, and the author takes care to mention the defects as well as the virtues of the technique adopted. In every respect the new journal has made an auspicious beginning, and is in our judgment already assured of success.

### THE IMPORTANCE OF A NAME.

That the name of a hospital may adversely affect its revenue is believed to be the case by the managers of the J. Hood Wright Memorial Hospital of New York, who have recently applied for and received permission from the Supreme Court to change the name of the institution. The reason for this change is that the name has given rise to the impression, which is an erroneous one, that the hospital is an endowed institution, and therefore the public have not come forward with the necessary financial support. The institution will in future be known as the Knickerbocker Hospital. The term infirmary is sometimes mistaken to imply rate support in this country, and we can recall an instance or two where the name has been actually changed on this account.

### COLLECTING FOR BOGUS CHARITIES.

At the holiday season it is especially necessary to be upon one's guard against the organisers of and the collectors for bogus charities, who, in spite of the often commendable action and restrictions of the town authorities, infest a great many of our larger seaside resorts. Although the evil is not so prevalent as it formerly was, owing to prosecutions having been from time to time instituted, it still, unhappily, exists. The collectors, often women or young girls or mere children, are very persistent, and some of them are attractive in appearance, and dressed in a respectable or tasteful manner, so that the good-natured holiday-maker cheerfully contributes his or her mite. Sometimes the so-called charities are of an altogether bogus character, and some of the collectors themselves are not aware, especially when they are young, that they are contributing to a fraud, for the organisers are artful and deceptive. Now and then there is "something to swear by," so to speak, a "holiday home" or something of the kind, but with no real claim to the title, and exploited on very doubtful principles. Anyhow, at this season of the year it is most necessary that due care should be exercised in bestowing our charity, and also that the outsides of the peripatetic collectors' boxes should be closely scrutinised to see what legends they bear.

### HOSPITAL PRESIDENT'S ROMANTIC SEARCH.

A very curious bequest has found its way at last to the Wirksworth Cottage Hospital, in Derbyshire, thanks to Mr. F. C. Arkwright, of Willersley Castle, the president of the institution. Mr. Arkwright, whose ancestor was the well-known inventor of the spinning jenny, which was the occasion, if not the cause, of the industrial revolution, still in progress, discovered that the present branch of the Capital and Counties Bank in Wirksworth, which used to be known as the Arkwright Bank, possessed—if that is the right word to describe an unconscious holding—a sum of money left to charity by the inventor's son which had not been found for use since 1843. Owing to Mr. Arkwright's instructions a search was made which disclosed a sum of £500 in Consols, two sums of £415 and £267 respectively, or a total in the bank of £1,182, which has been handed over to the governors to invest on the hospital's behalf. The rummage also produced another sum of £69, which was found in the will of Mr. Peter Arkwright, who died in 1866, and this has been added by the executors to the above bequest as a donation to the hospital. Few hospital presidents can have had such a romantic and satisfactory find.

### THE REPETITION OF PRESCRIPTIONS.

A Bill has been introduced in the House of Lords by Lord Lamington which proposes that where a medicine prescribed by a doctor contains a scheduled poison the prescription is not to be dispensed more than once unless the doctor gives directions either specifying the number of times it may be dispensed and supplied to the patient, or stating that it may be supplied to him or her as often, or in such quantities, as is desired. Any chemist who disobeys the instructions of the prescriber would be liable to a fine, for the first offence, not exceeding £10, and for the second or subsequent offence, to a fine not exceeding £50, and any person making a false statement for the purpose of obtaining medicine would be liable to three months hard labour. Some means of preventing the undue repetition of prescriptions are desirable, and are, in fact, provided in many countries, but it is doubtful whether Lord Lamington's stringent proposals will find favour. In hospital practice the difficulty seldom arises, and in private practice it would be quite sufficient for the prescriber to indicate on his prescription the number of times he wished it to be dispensed; there is no need to threaten the chemist with penalties, for chemists are only too anxious to retain the good will of medical practitioners.

### MORE MONEY FOR PRISON PHARMACISTS.

We understand that the Prison Commissioners, whilst favourably considering a revision of salary for prison officers, have granted the pharmacists in the service an increase of about 5 per cent. The salary will now start at £109 4s. and finish at £156, in addition to certain emoluments valued at about £25 per annum. The advance, which is given largely on account of the increased cost of living, brings the prison service into line with the other public bodies, mental and Poor Law, which have recently granted an increase.

### THE DOGS (PROTECTION) BILL.

The object of this measure, introduced and promoted with great energy by Sir F. G. Banbury, was to exempt dogs from acts of vivisection, while allowing the continuance of such acts upon other animals. This principle has been advocated especially also by Mr. Galsworthy, the dramatist, upon grounds of sentimentality; and undoubtedly it appeals to the emotional side of many people's natures. Unfortunately for Mr. Galsworthy, his *argumentum ad hominem* was knocked into a cocked hat by Mr. W. S. Lilly, another supporter of the Bill. There can be no doubt that these proposals were strongly supported by anti-vivisectors on thin end of the wedge grounds; and that numerous sentimentalists who see that the case for vivisection is too strong to be resisted supported the measure without realising what a powerful weapon it would place in the hands of anti-vivisectors. However, the Standing Committee of the House of Commons mutilated the Bill in such a way as to make it powerless to effect any real harm, and the promoters have therefore dropped it for the present.

### THIS WEEK'S DRUG MARKET.

Business in the Drug market has brightened up since the holiday, and several articles are attracting attention. A drop in the price of opium has taken place in the primary market, but the price movements of this drug are so uncertain that the value may advance again; the statistical position, however, would seem to justify lower prices, and cautious buyers are not in a hurry to make their purchases. There has been no further change in quotations for quinine, but any alteration is more likely to be in an upward than a lower direction. The price of citric acid is well maintained. Business has been done in ipecacuanha at slightly higher rates. Sugar of milk is tending dearer. The advance in the price of essence of lemon has received a check, and the tendency is towards lower values. The prospects of the English medicinal herb crops are, with some exceptions, fairly good; henbane was a good average crop, but belladonna is poor; peppermint, rosemary, and lavender are good, but the reports from France in regard to lavender are less favourable; aconite is disappointing.

### TO OUR READERS.

Contributions are specially invited from any of our readers to these columns. They should deal with topical subjects and news. They must be authenticated for the information of the Editor only. The minimum payment if published is 5s. There is no hard-and-fast rule as to space, but notes of about twenty lines in length are preferred.

## Figures and Tables

**Figure f1:**
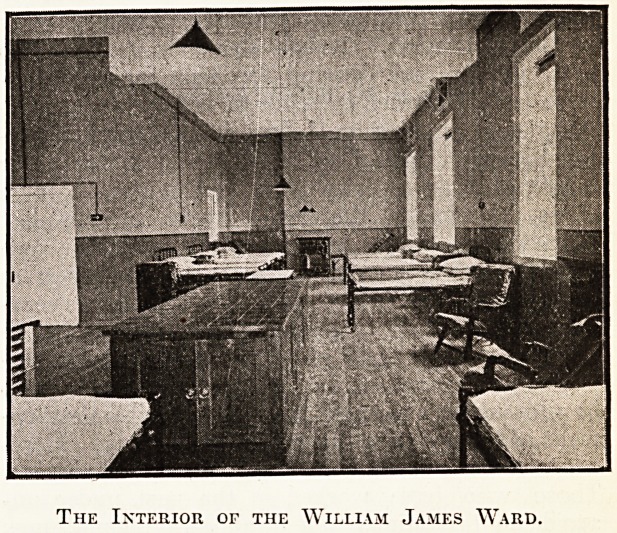


**Figure f2:**